# Supplementary material for: Monolithic integration of embedded III-V lasers on SOI
Source: Light Sci Appl. 2023 Apr 3;12:84. doi: 10.1038/s41377-023-01128-z (PMC10068801; doi:10.1038/s41377-023-01128-z)
Supplement: Supplementary file 1 — Supplementary Information [file 41377_2023_1128_MOESM1_ESM.docx]

**Supplementary Information**

**Monolithic Integration of Embedded III-V Lasers on SOI**

Wen-Qi Wei^1,2†^, An He^3†^, Bo Yang^1†^, Zi-Hao Wang^1,2,4†^, Jing-Zhi Huang^1,4^, Dong Han^1^, Ming Ming^1^, Xuhan Guo^3,*^, Yikai Su^3,*^, Jian-Jun Zhang^1,2,4,*^, Ting Wang^1,2,4,*^

^1^Institute of Physics, Chinese Academy of Sciences, Beijing, China

^2^Songshan Lake Materials Laboratory, Dongguan, Guangdong, China

^3^State Key Laboratory of Advanced Optical, Communication Systems and Networks, Department of Electronic Engineering, Shanghai Jiao Tong University, Shanghai, China

^4^School of Physical Sciences, University of Chinese Academy of Sciences, Beijing, China

*Corresponding authors: [guoxuhan@sjtu.edu.cn, yikaisu@sjtu.edu.cn](mailto:guoxuhan@sjtu.edu.cn,%20yikaisu@sjtu.edu.cn), [jjzhang@iphy.ac.cn](mailto:jjzhang@iphy.ac.cn), [wangting@iphy.ac.cn](mailto:wangting@iphy.ac.cn)

^†^These authors contribute equally to this work.

**I. Design and properties of edge couplers**

**
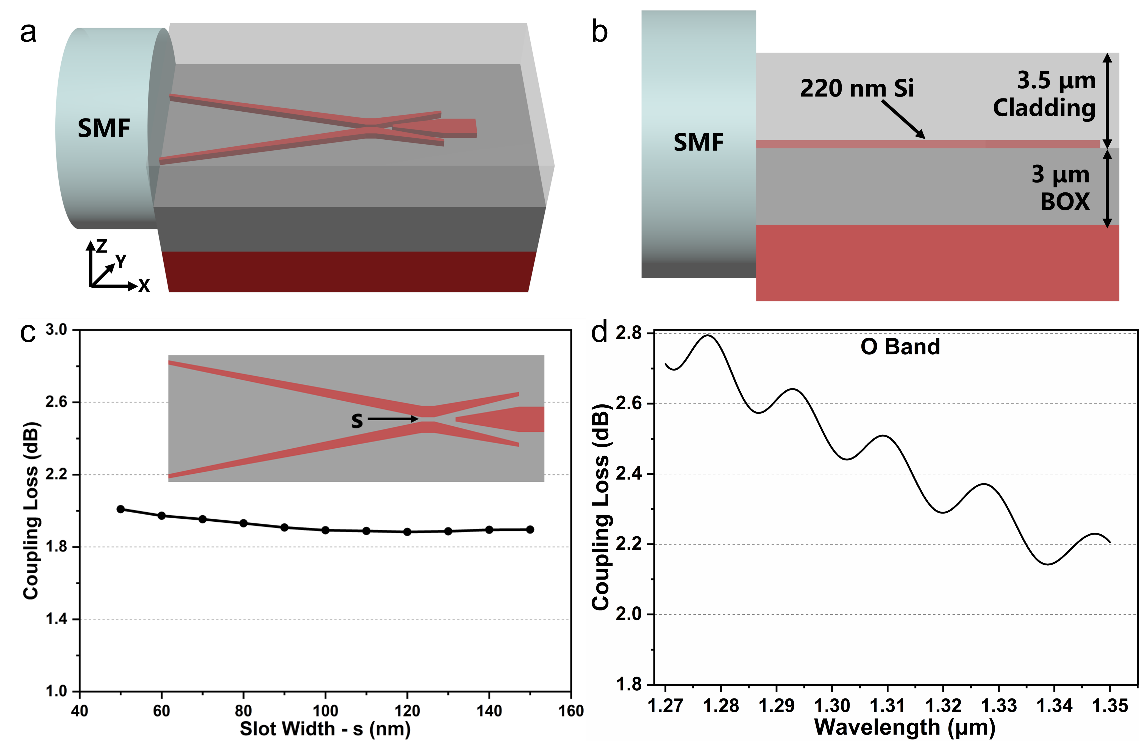
**

**Fig S1. The simulation results of the edge coupler used in the experiment.** **a** The edge coupling strategy from a single mode fiber (SMF) to the fork-shape edge coupler used in the experiment. **b** Cross-sectional schematic of the coupler, which is based on SOI platform with 3 μm BOX layer and 3.5 μm SiO_2_ cladding layer. **c** The coupling loss of SMF to chip facet versus slot width of the coupler. **d** Wavelength dependent coupling loss variations of SMF to chip facet.

For achieving high coupling efficiency and better alignment tolerance between the embedded ridge laser and the silicon strip waveguide, fork-shape edge couplers with double symmetrical tips (Fig. S1a and b) were used in the experiment^1, 2^. In order to examine the tolerance of edge coupler to large spot size light source, the simulation results of the coupling loss between the SMF and the coupler facet were performed in Fig. S1(c) and (d), with the change of slot width and laser wavelength. Slight variation (<0.2 dB) in coupling loss can be observed with the increase of slot width, which indicates a good fabrication tolerance of the coupler. Approximately 2.41 dB coupling loss between the SMF and the fork-shape coupler facet can be achieved, which is smaller than that of SMF/single-tip edge coupler^3^.


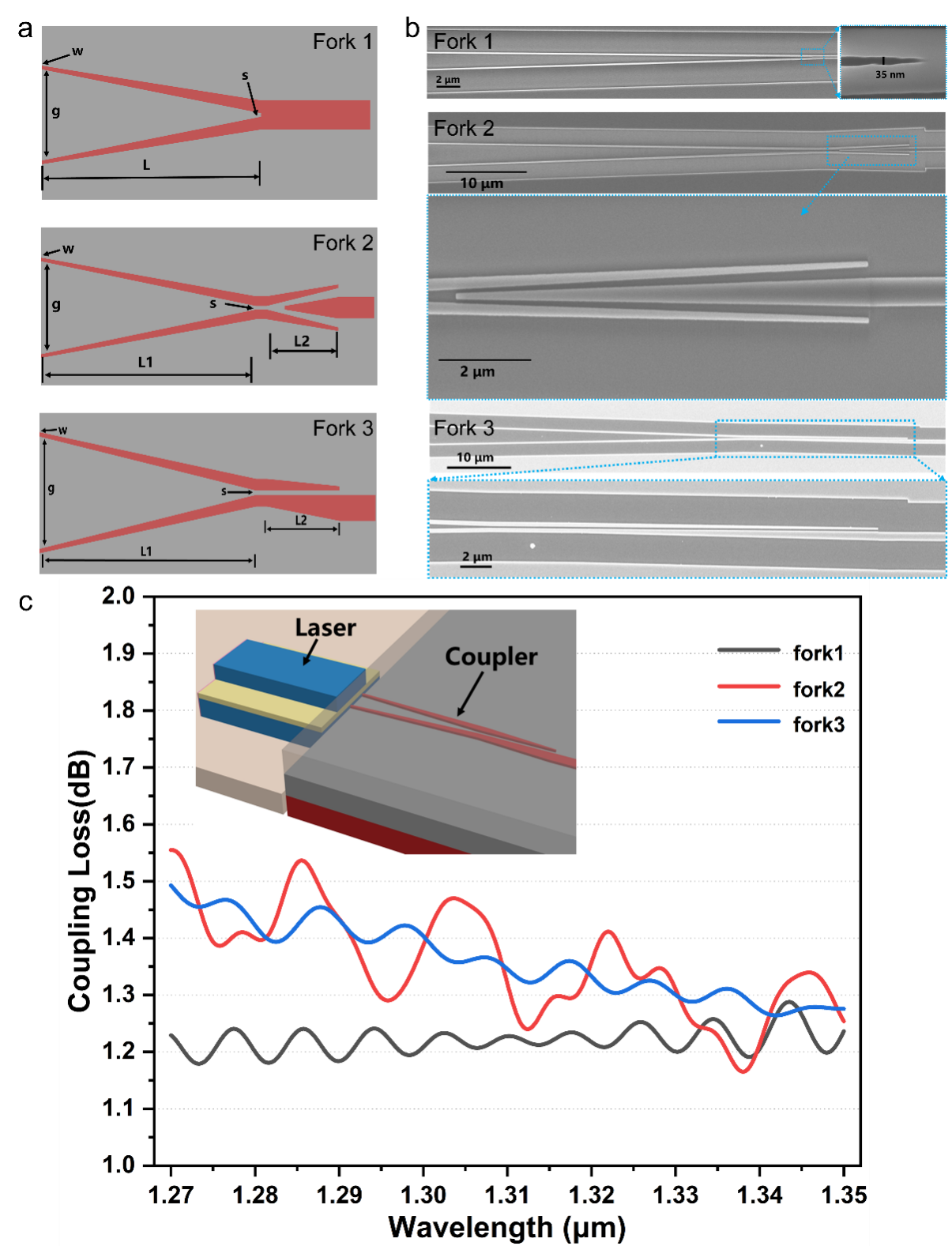


**Fig S2. The simulated coupling losses between laser and edge couplers.** **a** Schematics of the three different types of fork-shape edge couplers. **b** Top-view SEM images of fabricated three fork-shape edge couplers. **c** The simulated coupling losses in O-band among three different fork-shape couplers (shown in Fig. S2a) and III-V FP lasers. Inset: the edge coupling strategy from FP laser to the fork-shape edge coupler.

Furthermore, Fig. S2a shows the schematics of three different fork-shape edge couplers which are named as Fork 1, Fork 2, and Fork 3. Fig. S2b presents the top-view scaning electron microscope (SEM) images of the three fabricated fork-shape edge couplers shown in Fig. S2a. The simulated coupling losses between the three different fork-shape couplers and III-V fabry-perot (FP) lasers are shown in Fig. S2c. A minimum coupling loss of 1.18 dB can be obtained for Fork 1 with single symmetrical fork design, similar coupling loss f approximately 1.25 dB can be achieved for Fork 2 with double symmetrical fork design, which was used in the experiment. The coupling loss of 1.35 dB can be observed for Fork 3 with single asymmetrical fork design. These simulation results presented here provide precious guidance for fabricating high-quality edge couplers with low coupling loss used in monolithic integration of the embedded III-V QD laser edge-coupled with silicon waveguides in future experiment. Most importantly, the fork-shape edge coupler appears to be much more efficient for flat mode (semiconductor lasers) rather than spherical mode (SMF).

**II. Template design and epitaxial growth**

1. **Trenched SOI template for III-V growth**


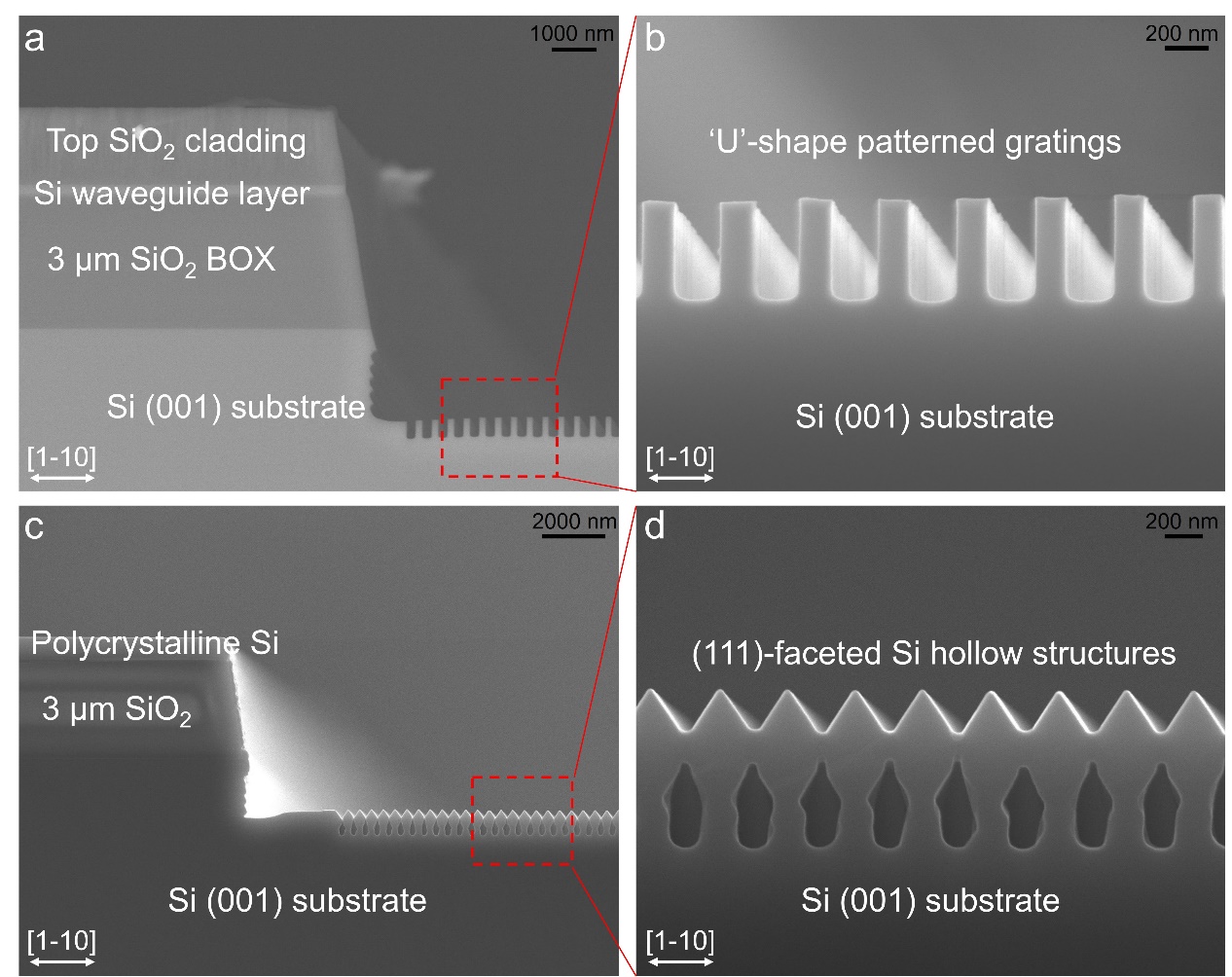


**Figure S3. Trenched SOI template for monolithic integration III-V lasers with silicon waveguides.** **a** Cross-sectional SEM image of the trenched SOI substrate with pre-defined silicon waveguide and “U”-shape patterned grating structures along [110] direction with a period of 360 nm. **b** Zoomed-in SEM image of the “U”-shape patterned grating structures in the trenched region marked in **a**. **c** Cross-sectional SEM image of the trenched SOI template after homoepitaxial growth of 420 nm silicon buffer layer. **d** Zoomed-in SEM image of (111)-faceted silicon hollow structures marked in **c**.

The SOI templates described in this work, with pre-defined waveguides on top silicon layer and trenched laser regions, are patterned with “U”-shape grating structures with 360 nm period (140 nm ridge width and 220 nm spacing width) along [110] direction (Fig. S3b) in the pre-patterned SOI trenches by electron beam lithography (EBL) and dry etching process as shown in Fig. S3a. The similar “U”-shape grating structures with same period and duty cycle have been utilized to grow high-quality III-V materials on both Si (001) and SOI substrates in our previous attempts. From our previous experience, both duty cycle and period of silicon gratings are relatively flexible in a range, which are typically 200 nm – 400 nm for the period and 30%-70% for the duty cycle. Here, silicon “U”-shape gratings should be fabricated close enough to the edge of SOI trench, in order to ensure reasonable edge-coupling efficiency between III-V laser and the silicon waveguide. The minimum coupling gap smaller than 3 μm can be achieved here as shown in Fig. S3a, by avoiding the edge effect during lithography. After homoepitaxial growth of 420 nm silicon buffer layer, the (111)-faceted silicon hollow structures are formed in the “U”-shape patterned region as shown in Fig. S3c and Fig. S3d. These structures are previously proved to be effective to suppress anti-phase domains (APDs), reducing threading dislocations (TDs) and thermal stress of the sample^4, 5^, while providing possibility for embedded epitaxial growth of on-chip III-V light sources.

1. **Embedded III-V epitaxial growth on trenched SOI substrate**

Early attempts on growing high-quality III-V materials in the trenched region on the SOI template were conducted to verify the feasibility of the design. The trenched SOI substrate with (111)-faceted silicon sawtooth structure was implemented for growth of III-V buffer layers as shown in Fig. S4a and Fig. S4b.

**
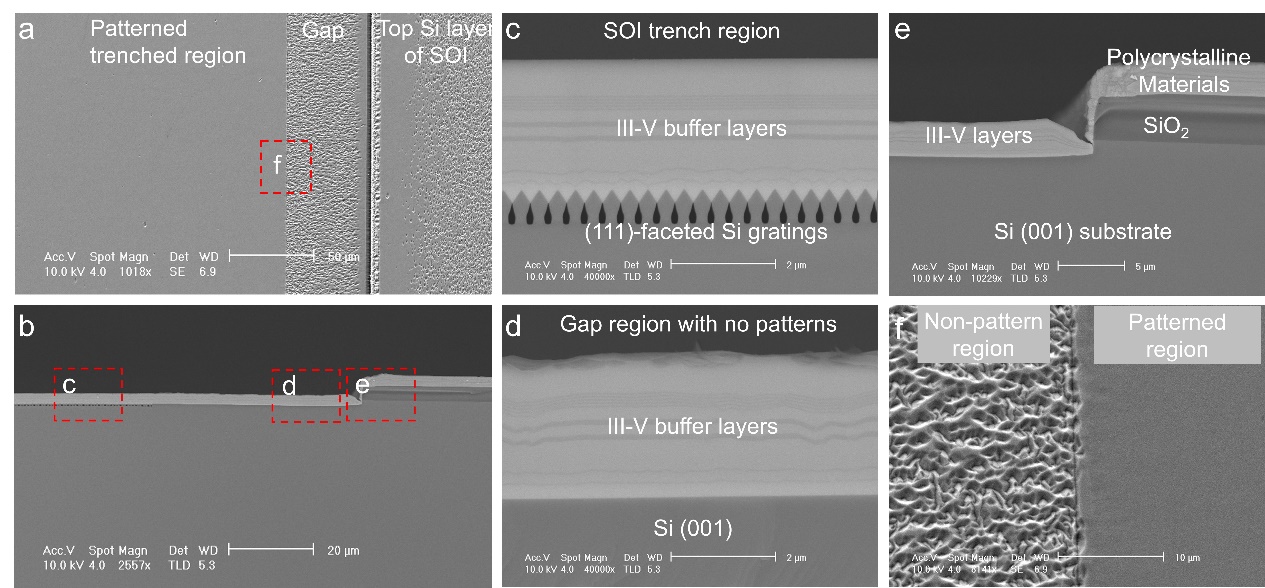
**

**Figure S4. III-V buffer layers grown on trenched SOI template.** **a** Top-view SEM images of III-V buffer layers grown on trenched SOI substrate with homoepitaxially formed sawtooth structures. **b** Cross-sectional SEM images of III-V buffer layers grown on trenched SOI substrate with homoepitaxially formed sawtooth structures. **c**, **d**, and **e** Cross-sectional SEM images of III-V layers grown on (111)-faceted silicon hollow structures, planer Si (001) and the edge of trenched region (the corresponding areas are marked in **b** ). **f** Top-view SEM image of the trenched region with and without patterned structures (the corresponding location is marked in **a**).

Fig. S4c and Fig. S4d show the cross-sectional SEM images of III-V buffer layers grown in the SOI trench region with silicon sawtooth structures and gap region without silicon sawtooth structures, respectively. The gap region, where there is no patterned structure, exhibit rough surface with large amount of dislocations (Fig. S4d). Fig. S4e shows the SEM image at edge of SOI trench, where there are significant amount of polycrystalline materials deposited on top silicon layer of SOI substrate. Fig. S4f displays the top-view SEM image of the trenched region with and without sawtooth structures marked in Fig. S4a, which presents APD-free III-V buffer layer with smooth surface grown in the patterned region of SOI trench. The success in epitaxy of III-V buffer layers on the trenched SOI substrate will support the following laser structure epitaxial growth on the specially designed template for monolithic integration of on-chip lasers and silicon waveguides described in this work.

1. **1300 nm InAs quantum dots (QDs) grown on the trenched SOI substrate**

**
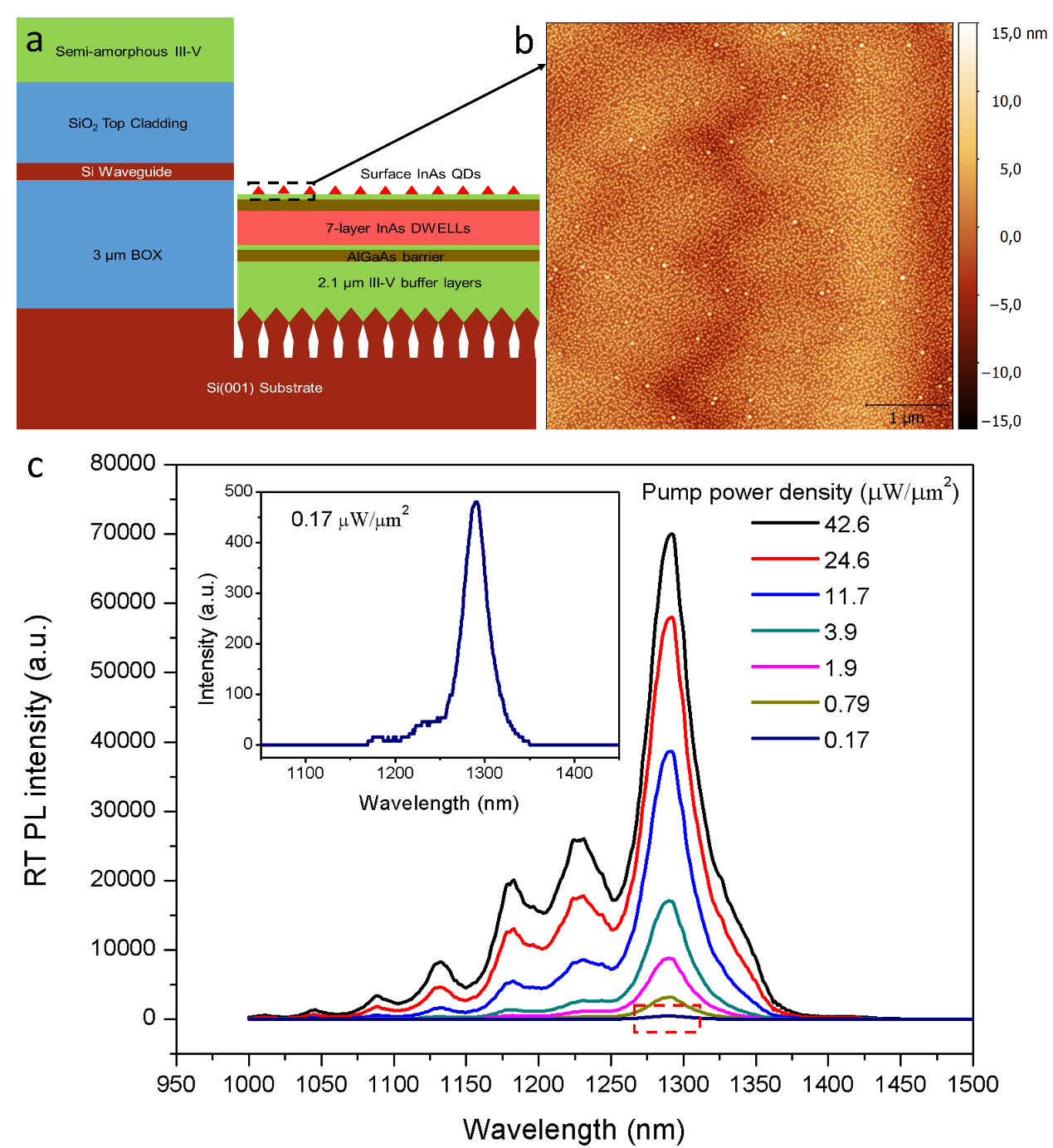
**

**Figure S5. 1300 nm InAs/GaAs QDs grown on the trenched SOI substrate.** **a** Schematic of a 7 layer of InAs/GaAs QD active region grown on the trenched GaAs/SOI substrate. **b** The 5×5 μm^2^ AFM image of the surface InAs QDs. **c** Pump power dependent room-temperature PL spectra of the InAs/GaAs QDs on the trenched SOI template. Inset: Zoomed-in PL spectrum of the sample at 0.17 μW/μm^2^ pump power density.

The optical properties of the InAs QDs grown on the trenched SOI substrate (reference sample) were also initially measured as shown in Fig. S5. Fig. S5a shows the schematic of the PL sample on trenched SOI substrate, which includes the 2100 nm thick III-V buffer layers and a 7 layer of InAs/GaAs QD active region. Fig. S5b displays the 5×5 μm^2^ AFM image of the surface InAs QDs on the trenched SOI substrate, indicating a good dot uniformity with a 5.1 × 10^10^/cm^2^ dot density. The defected dot density with a larger size can also be extracted with a value of 3.12 × 10^8^/cm^2^. Fig. S5c exhibits the pump power dependent room-temperature photoluminescence (PL) spectra of the sample. Notably, the excited-state like emission with multi-peaks can be observed from the PL spectra at a higher pump power density, which is caused by the cavity formed by the underlay grating structures and the smooth surface of the sample. This phenomenon has been witnessed in our previous work^4^. The inset in Fig. S5c shows the zoomed-in PL spectrum of the InAs QDs at 0.17 μW/μm^2^ optical pump power density, which presents a 1290 nm peak wavelength and a 31 nm FWHM of the spectrum. To note, the reference sample for PL measurements is different from the device sample in the manuscript in case of central wavelength due to slightly varied growth condition. The device sample exhibits relatively shorter central wavelength of 1270 nm, which shall not effect the overall performance.

**III. Characterization of the QD lasers monolithically integrated with butt-coupled silicon waveguides**

Light output power versus current (L-I) characterizations of the embedded III-V lasers under continuous-wave (CW) operation are measured in Fig. 5. Here, the pulsed injection currents (2 μs pulse width, 0.05% duty cycle) are applied as a comparison to evaluate the thermal effects of the SOI embedded QD lasers. For the single embedded laser without silicon WG (Fig. S6a), over 100 ^o^C operation temperature can be observed under pulse mode, while the maximum output power of 37.5 mW can be achieved under 275 mA current at 20 ^o^C. As Fig. S6b shows, the embedded laser coupled with silicon WG can also lase over 100 ^o^C, but has a maximum output power of only 6.5 mW under identical operation conditions, which is induced from the coupling loss between the laser facet and silicon coupler and the propagation loss of silicon WGs. The overall coupling loss can be estimated to be -7.6 dB under pulsed mode, which is similar to that under CW mode, indicating the thermal effect is minimum.

**
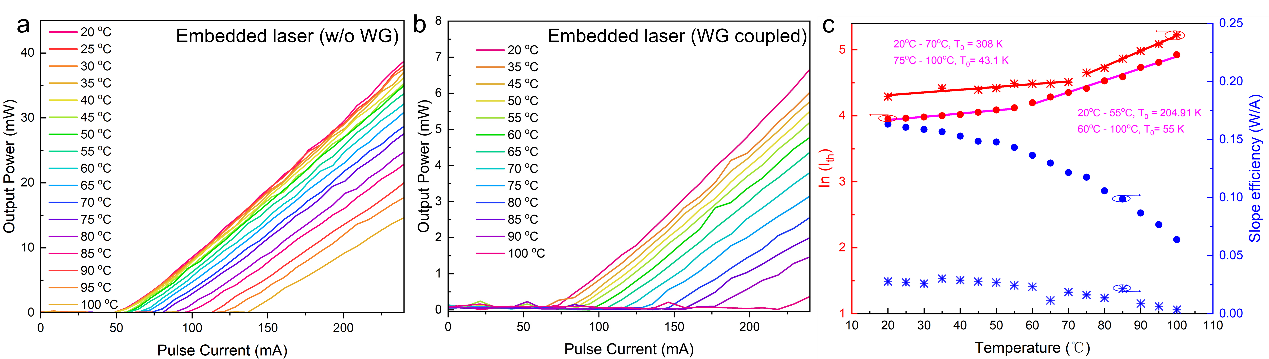
**

**Fig S6. Characteristics of the embedded III-V lasers under pulsed operation condition.** **a** and **b** The temperature-dependent L-I (light-current) curves of the embedded III-V lasers without and with coupled silicon waveguide, respectively. **c** Comparisons of the temperature dependent characteristic temperature (T_0_) and slope efficiency of the embedded III-V lasers with and without silicon waveguide.

Fig. S6c shows the temperature-dependent characteristic temperature (T_0_) and slope efficiency properties of on-chip lasers before and after coupling with silicon WG under pulse current mode. The SOI-based embedded laser itself owns the T_0_ values of 308 K (20 - 70 ^o^C) and 43.1 K (75 - 100 ^o^C). In comparison, the on-chip integrated laser coupled with silicon WG has slight degradation in T_0_ values, which are 204.91 K (20 - 55 ^o^C) and 55 K (60 - 100 ^o^C), due to surrounded waveguide materials. Furthermore, the single embedded laser itself shows a higher slope efficiency of 0.16 W/A at 20 ^o^C, while the embedded III-V laser with silicon WG presents that of 0.03 W/A at 20 ^o^C. Benefiting from the good heat dissipation of the substrate and the top thick metal contacts, the discrepancy in laser characteristics under CW and pulse current mode is negligible, which indicates that the SOI template implemented in this work can provide a reliable platform for monolithic integration high-performance III-V light sources with silicon waveguides. Furthermore, the temperature-dependent wall-plug efficiency for III-V laser on SOI with and without silicon waveguides are measured as shown in Fig. S7.


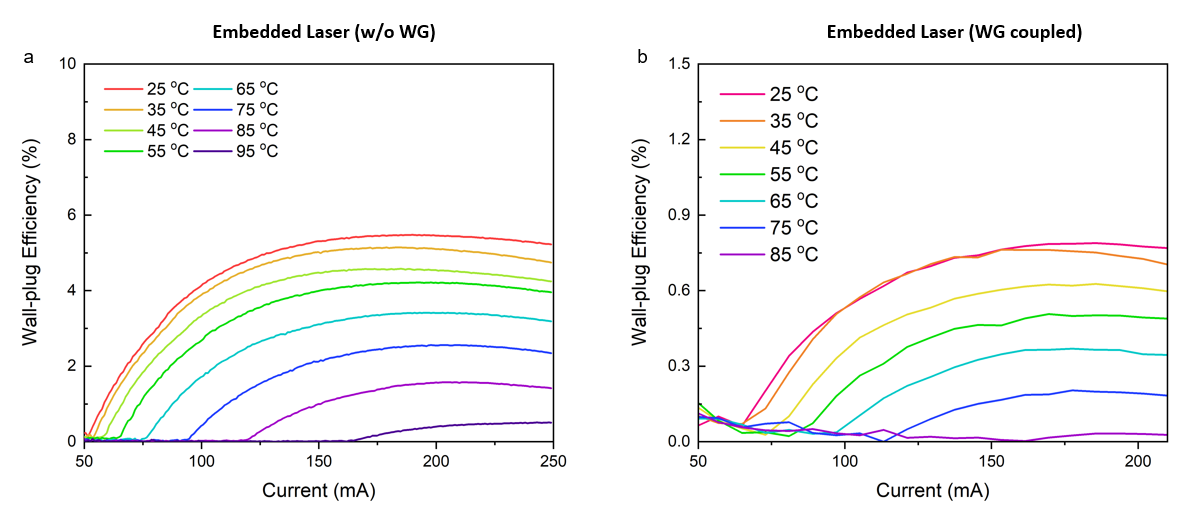


**Fig S7. Temperature-dependent wall-plug efficiency of the embedded III-V lasers under CW current operation condition.** **a** Embedded III-V laser on SOI without silicon waveguide. **b** Embedded III-V laser on SOI with monolithically integrated silicon waveguide.

**Reference**

1. He, A. et al. Low loss, large bandwidth fiber-chip edge couplers based on silicon-on-insulator platform. *J. Lightwave Technol.* **38**, 4780-4786 (2020).
2. He, A. et al. Ultracompact fiber-to-chip metamaterial edge coupler. *ACS Photonics* **8(11)**, 3226-3233 (2021).
3. Wang, J. et al. Comparison and analysis on single-layer Si fiber-To-chip edge couplers with different taper tips. *IEEE Int. Conf. Gr. IV Photonics GFP 2016-Novem*, 44–45 (2016).
4. Wei, W. Q. et al. InAs QDs on (111)-faceted Si (001) hollow substrates with strong emission at 1300 nm and 1550 nm. *Appl. Phys. Lett.* **113**, 053107 (2018).
5. Wei, W. Q. et al. Reliable InAs quantum dot lasers grown on patterned Si (001) substrate with embedded hollow structures assisted thermal stress relaxation. *J. Phys. D: Appl. Phys.* **55**, 405105, (2022).
